# Mitochondrial DNA mutations in ageing and cancer

**DOI:** 10.1002/1878-0261.13291

**Published:** 2022-07-28

**Authors:** Anna L. M. Smith, Julia C. Whitehall, Laura C. Greaves

**Affiliations:** ^1^ Wellcome Centre for Mitochondrial Research, Biosciences Institute Newcastle University UK

**Keywords:** ageing, cancer, metabolism, mitochondria, mitochondrial DNA, oxidative phosphorylation

## Abstract

Advancing age is a major risk factor for malignant transformation and the development of cancer. As such, over 50% of neoplasms occur in individuals over the age of 70. The pathologies of both ageing and cancer have been characterized by respective groups of molecular hallmarks, and while some features are divergent between the two pathologies, several are shared. Perturbed mitochondrial function is one such common hallmark, and this observation therefore suggests that mitochondrial alterations may be of significance in age‐related cancer development. There is now considerable evidence documenting the accumulation of somatic mitochondrial DNA (mtDNA) mutations in ageing human postmitotic and replicative tissues. Similarly, mutations of the mitochondrial genome have been reported in human cancers for decades. The plethora of functions in which mitochondria partake, such as oxidative phosphorylation, redox balance, apoptosis and numerous biosynthetic pathways, manifests a variety of ways in which alterations in mtDNA may contribute to tumour growth. However, the specific mechanisms by which mtDNA mutations contribute to tumour progression remain elusive and often contradictory. This review aims to consolidate current knowledge and describe future direction within the field.

## Graphical abstract

Mitochondrial DNA mutations are a common feature of ageing tissues and a number of human cancers. They can affect essential mitochondrial functions such as oxidative phosphorylation, redox balance, apoptosis, and numerous biosynthetic pathways. These are important mechanisms which reportedly impact tumour growth, though data are often contradictory. Here we review the current literature and describe future direction within the field.

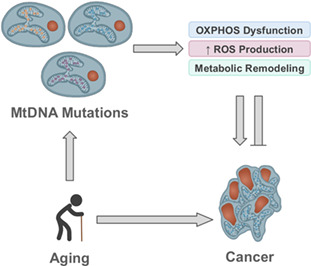

**Abbreviations**
CRISPR Cas9: clustered regularly interspaced short palindromic repeats and CRISPR‐associated protein 9
IMM: inner mitochondrial membrane
mitoDdCBEs: DddA‐derived cytosine base editors
mtDNA: mitochondrial DNA
mtZFNs: mitochondrially targeted zinc finger‐nucleases
NADH: nicotinamide adenine dinucleotide
OXPHOS: oxidative phosphorylation
PTEN: phosphatase and tensin homolog
ROS: reactive oxygen species
TCA cycle: tricarboxylic acid cycle
yPTP: yeast permeability transition pore

## 1 Introduction

### 1.1 Mitochondria

Mitochondria are dynamic, double‐membrane‐bound organelles, which are present in the cytoplasm of almost every eukaryotic cell. Mitochondria play an essential role in metabolism, converting carbohydrates and fatty acids into ATP, via oxidative phosphorylation (OXPHOS) [1]. Alongside this critical function, mitochondria are also involved in other functions including cytosolic calcium regulation [2], apoptosis [3] and the biosynthesis of heme and iron sulfur (Fe‐S) clusters [4]. Unlike other organelles, mitochondria contain their own genome, mitochondrial DNA (mtDNA). This circular, self‐replicating, double‐stranded molecule comprises 16 569 base pairs in humans and encodes 13 essential proteins of the OXPHOS system; the additional proteins required are encoded by the nuclear genome [5].

#### 1.1.1 The mitochondrial genome

**Fig. 1:**
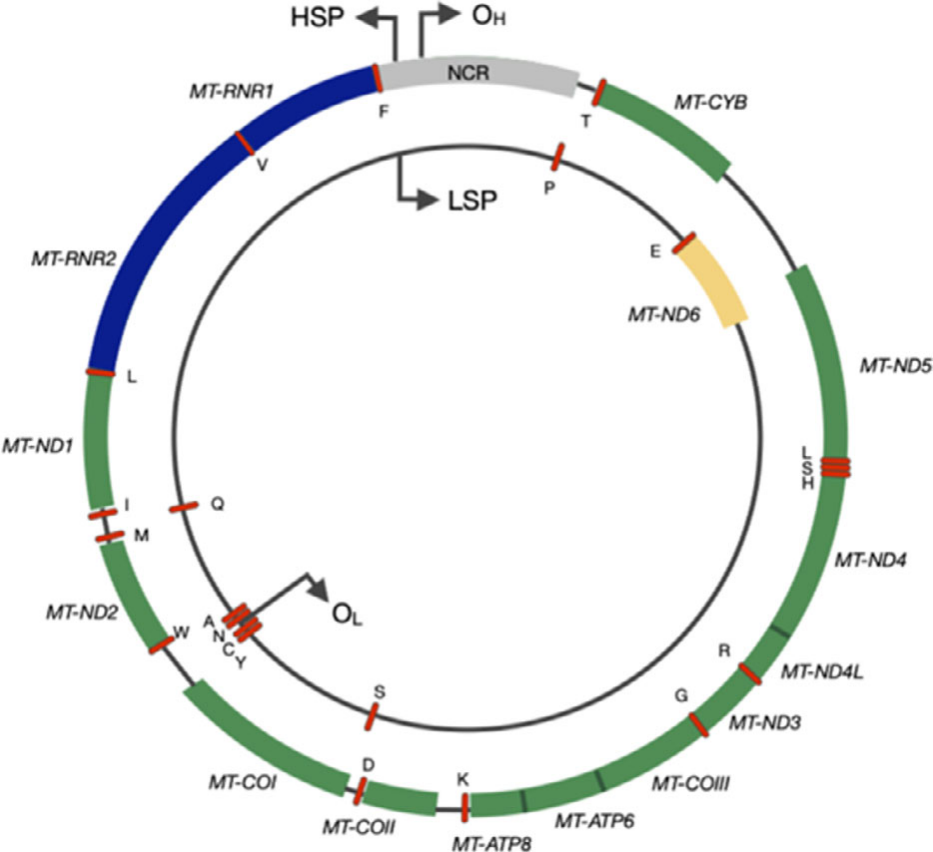
The human mitochondrial genome. The outer circle depicts the heavy strand with the inner circle representing the light strand. Genes encoding proteins of the mitochondrial respiratory chain are shown as coloured blocks labelled accordingly (*MT‐ND1–6*, *MT‐COI–III*, *MT‐ATP6* and *8* and *MT‐CYB*). The blue blocks denote the two ribosomal RNA and red dashes represent each of the 22 tRNA. [Colour figure can be viewed at wileyonlinelibrary.com]

The mitochondrial genome encodes 37 genes of which 22 are transfer RNA (tRNA), two are ribosomal RNA (12S and 16S ribosomal subunits), and the remaining 13 encode polypeptides involved in OXPHOS. The mitochondrial genome is very compact, lacking introns, and contains only one major noncoding regulatory region, the D‐loop (Fig. 1). In contrast to the nuclear genome which is packaged into nucleosomal structural units by association with histones, mtDNA lacks histone support and is instead organized into discrete units known as nucleoids which the earliest reports describe as containing one or two mtDNA molecules [6]. Nucleoid organization ensures the appropriate distribution of mtDNA throughout the mitochondrial network, and core components have been proposed to comprise factors which are involved in mitochondrial replication and transcription such as mitochondrial transcription factor A (TFAM) [7], mitochondrial DNA polymerase gamma (Polγ), mitochondrial RNA polymerase (POLRMT), mitochondrial single‐strand binding protein (mtSSB) and the mitochondrial helicase TWINKLE [8]. Peripherally, proteins such as ATPase AAA domain‐containing protein 3 (ATAD3) and the prohibitins 1 and 2 (PHB1 and PHB2) facilitate inner mitochondrial membrane tethering while also mediating ribosomal contact and thus supporting protein synthesis [9]. The abundance of TFAM and thus the level of DNA compaction have been shown to impact the proportion of mtDNA molecules available for transcription or replication [10]. Nucleoid organization therefore supports mitochondrial form and function.

#### 1.1.2 Heteroplasmy and the threshold effect

**Fig. 2:**
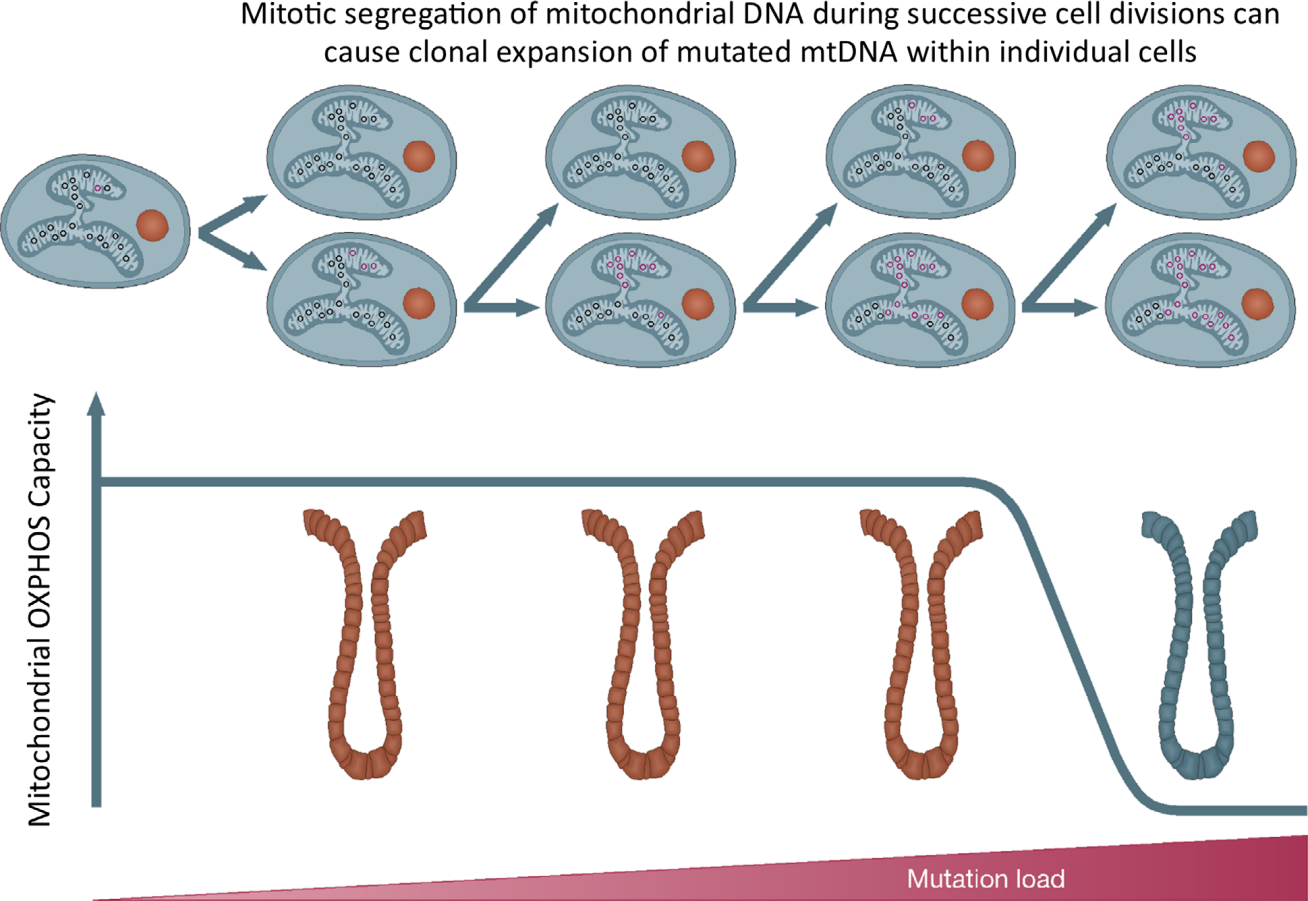
Clonal expansion of mtDNA mutations through mitotic segregation. Somatic mitochondrial DNA (mtDNA) mutations occur randomly primarily through replication errors, as well as mis‐incorporation of incorrect bases opposite oxidative adducts. Upon cell division, mtDNA molecules randomly segregate to daughter cells and are then replicated to maintain consistent mtDNA copy number. This could either result in loss of the mutation or clonal expansion within that cell lineage. Through multiple successive rounds of cell division and vegetative segregation, the mutated mtDNA molecules can become the dominant species within the cell. When the critical threshold for mutated mtDNA is reached, the wild‐type molecules are no longer able to compensate and a biochemical defect in oxidative phosphorylation (OXPHOS) occurs. A schematic of the colonic epithelium is shown here, and brown crypts represent those with low‐level mtDNA mutations and normal OXPHOS activity. The blue crypt represents a crypt in which the mtDNA mutation load has crossed the threshold and has a measurable OXPHOS deficiency. [Colour figure can be viewed at wileyonlinelibrary.com]

Hundreds to thousands of copies of the mitochondrial genome are present within each cell. Homoplasmy describes the state in which all copies of mtDNA are identical, whereas heteroplasmy refers to the state in which there is more than one mtDNA variant in a cell. [11, 12]. The majority of mtDNA mutations are functionally recessive, and when present at low levels, functional complementation occurs, enabling the cell to retain OXPHOS capacity. It is only when a critical threshold of mutant mtDNA copies is reached that a biochemical defect becomes manifest (Fig. 2) [11]. This threshold can differ for different mutations, for example, the common mtDNA disease‐causing point mutations m.3243A>G and m.8344A>G must typically reach a mutation load of 80–90% before OXPHOS function is compromised [13], whereas large‐scale mtDNA deletions are typically associated with a threshold level of approximately 60% [14].

#### 1.1.3 Clonal expansion

Clonal expansion describes the process by which a mutated molecule of mtDNA accumulates within a cell over time to become the dominant species. At present, it is unclear exactly how this occurs; however, there are several mechanistic theories. The first is based upon the theory that copies of mtDNA harbouring large‐scale deletions have an advantage over wild‐type molecules due to their smaller genome taking less time to replicate [15]. This is known as the ‘survival of the smallest’ theory and has been supported by experimental evidence both *in vitro* [16] and *in vivo* [17]. However, with regards to point mutations, which are most commonly detected in both normal mitotic tissues [18, 19, 20] and tumours [21], the genome size is unaffected. This mechanism is therefore unable to account for expansion of these clones.

The mechanism that most likely explains the clonal expansion of mtDNA mutations in rapidly dividing cell populations such as tumours is random genetic drift and mitotic segregation. Replication of the mitochondrial genome occurs independently of the cell cycle; however, mitochondrial dynamics and morphology coordinate with particular cell cycle stages in order to facilitate the partitioning of mitochondria to daughter cells at cell division. Prior to division, the mitochondrial genome must replicate to ensure sufficient copies are transmitted to daughter cells. Between G1 and S phases, the fusion of mitochondria is promoted, and the network can be visualized as an extensive, hyperfused system [22]. In the early stages of mitosis, the phosphorylation of the fission promoting dynamin‐related protein 1 (DRP1) results in fragmentation of the mitochondrial network prior to their segregation as the cell divides [23]. As the segregation of mitochondria at mitosis is a stochastic process, if there is a heteroplasmic variant present within the parent cell, daughter cells have equal chances of acquiring more, less, or the same level of mutant mtDNA molecules as the cell from which they are descended. Accordingly, mitotic segregation may inadvertently contribute to, and potentially accelerate, the clonal expansion of mutant clones as has been demonstrated using simulated modelling within normal buccal epithelial cells, tumour cells [24] and more recently within colonic crypts [25] (Fig. 2).

## 2 Mitochondrial DNA mutations and ageing

The ageing process has been described as the accumulation of molecular and cellular damage that results in reduced function in adulthood, diminished fecundity and an increased probability of death [26]. One common feature of human ageing is an accumulation of cells with mitochondrial dysfunction. The accumulation of mtDNA mutations was first described in postmitotic tissues such as cardiomyocytes, skeletal muscle and brain around three decades ago [27, 28, 29]. The accumulation of mtDNA deletions and resultant OXPHOS defects within human aged substantia nigra neurons has been strongly associated with age‐related neurodegeneration and Parkinsonism [30, 31]. Regarding skeletal muscle, an elegant study measured the mtDNA deletion level in cytochrome *c* oxidase (COX)‐deficient regions of serially sectioned rat skeletal muscle fibres. They showed that the deletions were at the highest levels of heteroplasmy in the COX‐deficient regions and that these were the points at which breaks and splits of the fibres were occurring [32]. Combined, these studies show that mtDNA deletions can have an influence on major aspects of cellular physiology in ageing postmitotic tissues.

Focusing in this review on mitotic tissues, the first documented evidence of mtDNA mutations in such tissues was the detection of clonally expanded mtDNA point mutations in single aged buccal epithelial cells [33]. Subsequently, single‐cell mtDNA sequencing of laser microdissected COX‐deficient crypts of the aged human colon supported the work of Nekhaeva et al. [18]. The presence of single mutations in entirely COX‐deficient crypts, the majority of which were transitions involving G residues, implied the expansion of stem cell‐derived mtDNA mutations from parent stem cells to all progeny. Additional studies have shown that this is not exclusive to the buccal or colonic epithelium; OXPHOS defects and mtDNA point mutations have been detected in a variety of tissues with mitotic capability such as the liver [20] and the stomach [19]. Understanding the mechanisms by which mitochondrial dysfunction can promote the ageing phenotype is difficult in isolated human tissues but may have important implications in the treatment or even the prevention of age‐related diseases. To this end, the development of two similar mouse models in 2004 and 2005 allowed the potential causative effect of mtDNA mutation accumulation on the ageing process to be investigated [34, 35]. Both models share the same amino acid substitution at the same conserved residue within the mitochondrial *PolγA* gene. An aspartate to alanine substitution at residue 257 of the conserved proofreading domain of *PolγA* impairs the exonuclease activity of the enzyme resulting in an up to eightfold increase in the likelihood of point mutations occurring during replication of the mitochondrial genome [35]. The random cumulative generation of mtDNA point mutations, and their subsequent cellular clonal expansion, results in a premature ageing phenotype with greying of hair, curvature of the spine, reduced subcutaneous fat, sarcopenia, osteoporosis, reduced fertility, anaemia and heart enlargement leading to a significantly reduced lifespan (< 12 months). Subsequent studies have shown that the accumulation of mtDNA point mutations in the stem cell compartments of the *PolγA* ^*mut/mut*^ mice is likely to underlie the premature ageing phenotype [36, 37, 38, 39]. These were the first studies to suggest a ‘causative’ link between mtDNA mutations and ageing.

The ability of the *PolγA* ^*mut/mut*^ mouse to accurately model human ageing has, however, been challenged. Firstly, the level of mtDNA mutation is much higher in the mutator mouse than in aged human tissues [40], and despite a significant increase in mtDNA mutation load, the *PolγA* ^*+/mut*^ mouse does not present with a premature ageing phenotype. This has led some groups to argue against a role for mtDNA point mutations in the pathology of normal ageing [41]. Despite the reported absence of an overarching progeroid phenotype in the *PolγA* ^*+/mut*^ mouse, analysis of the small intestine of 15‐month‐old mice identified mtDNA point mutations with high levels of heteroplasmy within COX‐negative villi [42]. OXPHOS defects were also detected in the colon at 12 [38] and 17 months [43]. The frequency of OXPHOS deficiency in these tissues was more similar to aged humans than the *PolγA* ^*mut/mut*^ mice [43], and as the *PolγA* ^*+/mut*^ mice have a normal lifespan, they may have significant utility in deciphering effects of mosaic cellular OXPHOS defects alongside other mechanisms of normal ageing.

The second challenge is around the somewhat contradictory data on the role that mtDNA deletions play in the progeria observed in the *PolγA* ^*mut/mut*^ mice. Vermulst et al. reported an accelerated age‐related increase in the frequency of mtDNA deletions in the brains of *PolγA* ^*mut/mut*^ mice in comparison with *PolγA* ^*+/mut*^ and wild‐type mice [42]. They proposed that mtDNA deletions rather than point mutations were the driving force behind the advanced ageing phenotype seen in *PolγA* mutator mice [42]. This accumulation of canonical deletions was only observed in the mice developed by the Prolla group [35], and similar analyses of tissues in the mice developed by the Larsson group [34] detected no canonical circular deletions [44], suggesting that different mutational mechanisms are driving the ageing phenotype in the two strains. This puzzling discrepancy is as yet unresolved. However, there are differences between the background strain of the mice (the Prolla mice are on a C57Bl6/J background, and the Larsson mice are on a C57Bl6/N background), and there are differences in the transgenic construct used to generate the two lines, which might explain the contradictory findings of the two groups. Bringing all of these data together, while it is clear that the accumulation of both mtDNA point mutations and deletions is a feature of normal human ageing, and at extreme levels, they can cause progeria in mice, and there is still no consensus on their contribution to normal ageing. It is likely that the functional consequences of these mutations and their role in the pathophysiology of ageing are going to be dependent on the cell type, the tissues in which they reside and may even be species specific.

The consequences of mtDNA mutation accumulation with age extend beyond the impairment of OXPHOS and the functional decline that this elicits. For example, mitochondrial dysfunction and reactive oxygen species (ROS) production facilitate telomere shortening and promote cellular senescence, both additional hallmarks of ageing [45], highlighting the multifaceted role of mitochondria in ageing.

## 3 Mitochondria and malignancy

Almost 100 years ago, Otto Warburg postulated that tumour cells preferentially utilize aerobic glycolysis as their primary means of energy production even in the presence of sufficient oxygen [46]. In terms of ATP production, glycolysis represents a much less efficient system yielding two molecules of ATP per molecule of glucose in comparison to the net 36 molecules generated via mitochondrial OXPHOS. Although in part compensated for by the increased expression of glucose transporters and increased glucose uptake, a predominantly glycolytic metabolism bestows tumour cells with a greater abundance of glycolytic intermediates available for diversion into biosynthetic pathways such as the pentose phosphate pathway [47, 48]. Key enzymes of the pentose phosphate pathway are commonly overexpressed in cancer, and this aids the generation of ribose sugars required for nucleic acid synthesis and also NADPH, which supports fatty acid synthesis and antioxidant defence (reviewed in ref. [49]). Similarly, the diversion of fructose‐6‐phosphate into the hexosamine pathway has been shown to provide substrates required for glycosylation of proteins contributing to increased proliferation and progression of colorectal cancer [50]. The primary growth and proliferation demands of the tumour are thus facilitated by a glycolytic shift; however, it is becoming apparent that this shift is not an event that occurs passively but as a result of active reprogramming driven by the tumour itself (reviewed in ref. [51]). This is exemplified by members of the MYC family of transcription factors, which are commonly deregulated and oncogenic in many human cancers. MYC has been shown to promote tumour anabolism via an upregulation of the glucose transporter protein type 1 (GLUT1) and an increase in the expression of glycolytic enzymes such as glyceraldehyde‐3‐ phosphate dehydrogenase (GAPDH) and phosphofructokinase (PFK) [52]. Conversely, the tumour suppressor p53 can inhibit glycolysis via the downregulation of GLUT1/4 [53] and can regulate the balance between oxidative and glycolytic metabolism [54]. Additionally, in mice, the abundance of mutant copies of activating *Kras* has been shown to correlate with enhanced glycolysis and increased glucose‐derived TCA metabolite and glutathione production facilitating detoxification in non‐small‐cell lung cancer (NSCLC) [55]. In proliferating cells, precursors derived from TCA intermediates are frequently utilized as precursors for lipids, nucleic acids, protein synthesis and epigenetic modifications and can act as oncometabolites (Box 1).

Box 1 **Metabolites of the Tricarboxylic acid cycle can have downstream functions that modulate tumour behaviour through epigenetic modification or HIF‐1α stabilization**

This discovery was initially made following the finding of gain of function mutations in genes encoding both the cytoplasmic and mitochondrial isoforms of isocitrate dehydrogenase (*IDH1* and *IDH2*, respectively) in high‐grade gliomas and glioblastomas. These mutations result in a neomorphic enzyme activity [156, 157] where the usual catalytic interconversion of isocitrate and α‐ketoglutarate (α‐KG) by IDH1 and IDH2 is replaced by the reduction of α‐KG generating the (*R*)‐2‐hydroxyglutarate (*R*‐2HG) oncometabolite, which subsequently causes histone and DNA hypermethylation via its inhibition of α‐KG‐dependent demethylase enzymes [158, 159]. Similarly, loss of function mutations in additional TCA cycle enzymes fumarate hydratase (FH) and succinate dehydrogenase (SDH) can have pro‐tumorigenic consequences for the cell due to a build‐up of their substrates fumarate and succinate. The accumulation of oncometabolites fumarate and succinate, like *R*‐2HG, can give rise to widespread histone and DNA hypermethylation due to the inhibition of α‐KG‐dependent demethylases [160, 161]. Resulting from a loss of function mutation in FH, the accumulation of fumarate has also been shown to promote epithelial‐to‐mesenchymal‐transition (EMT) by inhibiting the demethylation of an antimetastatic miRNA cluster [162], while the loss of either FH or SDH and accompanying accumulation of substrates can further promote tumour survival via hypoxia‐inducible factor 1 (HIF‐1) stabilization‐mediated angiogenesis and glycolytic adaptation [163, 164]. Recently, an accumulation of fumarate in FH‐deficient renal cell carcinoma (RCC) has been shown to lead to an increase in mtDNA mutations and impaired respiratory complex activity due to the fumarate‐mediated succination of POLG, TWINKLE and TFAM proteins, thus promoting an irreversible shift to aerobic glycolysis and disease progression [165].

Since the early observation that tumour cells generate greater quantities of hydrogen peroxide than normal cells, mitochondrial ROS have been implicated in tumourigenesis [56]. Early work directly demonstrated that overexpression of the mitochondrial superoxide dismutase suppressed neoplastic transformation [57], while more recently the knockdown of genes involved in the regulation of autophagy predisposed cells to increased ROS, secondary mutations and transformation [58]. Further to their mutagenic potential, ROS are important mediators of both pro‐ and anti‐tumourigenic signalling. Hydrogen peroxide in particular has been shown to oxidize and inactivate the tumour suppressor, thus removing negative regulation of the PI3K/Akt/mTOR survival pathway [59], while KRAS‐induced mitochondrial ROS generation activates epidermal growth factor receptor (EGFR) signalling via the NF‐κB transcription factor and its ligands in pancreatic acinar cells [60]. The progression of malignancy to metastatic disease has also been demonstrated to involve regulatory input from ROS. Tyrosine kinases central to the cell adhesion response are reportedly activated by ROS, thus promoting anchorage independent growth of transformed cells [61]. Paradoxically, matrix detachment causes further increases in ROS production, which can subsequently inhibit distant metastases [62, 63]. Excessive oxidative stress can also promote apoptotic cell death via activation of the JNK and p38 signalling pathways [64]; therefore, in order to migrate and survive, tumour cells must employ antioxidant machinery to counteract such stresses. The delicate balance between pro‐ and anti‐tumourigenic ROS signalling is evidently key to tumour survival and as such highlights the central role of mitochondria in the tumuorigenic process.

### 3.1 mtDNA mutations in cancer

Contrary to Warburg's hypothesis that the aerobic glycolysis observed in tumour metabolism occurs on account of completely defective mitochondria, many tumours retain OXPHOS capacity and in fact tumourigenesis is inhibited in tumour cells completely depleted of mtDNA [65, 66]. However, mutations of the mitochondrial genome, which modulate OXPHOS function, have been reported in human cancers for decades. Initial reports in the late 1980s documenting the presence of mtDNA deletions within human renal oncocytomas [67] were followed by studies documenting D‐loop deletions within gastric adenocarcinomas [68], *MT‐ND1* deletions within renal cell carcinoma (RCC) [69] and large‐scale 4977 bp mtDNA deletions within breast cancer [70]. A number of studies subsequently reported the presence of clonally expanded somatic mtDNA point mutations in different tumour types. D‐loop mutations were identified in colorectal and gastric tumours [71], and mutations within the D‐loop and protein coding genes (primarily *MT‐ND4*) were reported within bladder, head and neck, and lung malignancies [72]. Within ovarian carcinomas, mutations within the 16S and 12S rRNA genes, the D‐loop and the MT‐CYB gene were most frequent [73]. In 1998, Polyak et al. were the first to detect somatic mtDNA point mutations at homoplasmic levels in seven out of ten solid human colorectal tumours [74]. These findings have been confirmed in a number of more recent studies [75, 76, 77]. Interestingly, the mtDNA mutational profile observed in colorectal cancers is also very similar to that observed within normal ageing colonic crypts [18, 78, 79]. Furthermore, the detection of pathogenic truncating mtDNA mutations at high levels of heteroplasmy and homoplasmy within colorectal cancers [80] implies that age‐related somatic mtDNA mutations are enriched as normal crypts transform into malignant lesions. However, there is also evidence that mtDNA mutations can reach high levels of homoplasmy within tumour cells without selection or enrichment and achieve this status passively through multiple rounds of mtDNA replication and cell division [24]. Using a computer simulation model, Coller and colleagues demonstrated that in as few as 70 rounds of mtDNA replication and random segregation at cell division, in the absence of any selective pressures, a predicted fraction of 0.2 homoplasmic mutant epithelial cells can reach the frequency of homoplasmic mutation reported in tumours. Overall, this mode of neutral drift devoid of selective pressures has been supported by large‐scale pan‐cancer genomic analyses of patient tumour and matched normal tissue samples [80, 81, 82, 83]. However, when these data are stratified by cancer type, cancers of the kidney, colon and thyroid actually display evidence of the positive selection of deleterious mtDNA mutants in complex I genes, but no selection on neutral variants, suggesting that these mutations may be advantageous only in those tissue‐specific contexts [21, 80, 81]. Conversely, the negative selection of severely disruptive truncating mutations within complex V genes was shown in all cancer types studied [21], suggesting that total loss of ATP synthase activity is not tolerated. These large‐scale pan cancer genomic analyses have been extremely useful in documenting the presence, prevalence and spectrum of mtDNA mutations in cancers. However, they do not tell us about the pathogenicity of these variants, either in terms of mitochondrial OXPHOS capacity or whether they have direct or indirect effects on tumourigenesis.

### 3.2 Cellular strategies to investigate functional effects of mtDNA mutations in cancer

As there is still no clear consensus on whether mtDNA mutations have tumour promoting [84], tumour suppressing [85] or neutral effects in carcinogenesis, researchers have turned to cell models to try and disentangle these often conflicting data. The earliest strategy was the generation of trans‐mitochondrial cytoplasmic hybrid (cybrid) cell lines [86]. Here, mitochondria with either wild‐type or mutated mtDNA are fused with nuclear donors devoid of mtDNA, to directly investigate the effect of different mtDNA genotypes in a defined nuclear background. [87]. Examples of such studies in various cancer lines are described in the later sections of this review. While trans‐mitochondrial cybrid models are widely used, as with all models of disease there are disadvantages which must be noted when considering their reliability. The generation of cell lines devoid of mitochondria has traditionally been achieved with the application of compounds which inhibit mtDNA replication such as ethidium bromide, a DNA intercalating dye [86] or dideoxycytidine, a nucleoside reverse transcriptase inhibitor. However, ethidium bromide in particular can be mutagenic to nuclear DNA with long periods of exposure, and the requirement for selection of clones following fusion can lead to significant intraclonal heterogeneity due to founder effects. Bearing this in mind, downstream analyses should always use multiple clones; however, this is rarely done in practice. Finally, the generation of cybrids excludes the innate nuclear‐mitochondrial cross talk potentially modifying cellular behaviour [88]. To generate cells with wild‐type and mutated mtDNA on an isogenic nuclear background, an alternative strategy was the modulation of a 143B osteosarcoma cybrid line with a heteroplasmic m.8993T>G mutation using mitochondrially targeted zinc finger‐nucleases (mtZFNs). mtZFNs degrade mutant mtDNA through site‐specific DNA cleavage and can be used to fine‐tune heteroplasmy levels without the need for clonal selection (termed mTUNE cells) [89]. Three lines were generated with heteroplasmy levels at 7%, 45% and 80% and were subsequently used to investigate the effects of m.8993T>G on cancer cell metabolism [90]. A disadvantage of both cybrids and mTUNE is the fact that the mtDNA mutations that can be modelled are limited to those that have arisen naturally, usually in cell lines from patients with primary mtDNA diseases, and there is no scope to model‐specific mtDNA mutations of interest if a cell line does not already exist. It is only very recently that site‐specific mutagenesis of the mtDNA has become a possibility. Driven by the inability of nuclear‐based gene editing technologies such as clustered regularly interspaced short palindromic repeats and CRISPR‐associated protein 9 (CRISPR Cas9) to be directed into the mitochondria, much effort has been made in the generation of novel gene‐editing tools which target mtDNA and do not depend on the CRISPR architecture. Two novel base editing tools, DddA‐derived cytosine base editors (mitoDdCBEs) [91] and TALE‐linked adenine deaminases (TALEDs) [92] capable of inducing site specific C>T and A>G mutations, respectively, have been developed (reviewed in ref. [93]). These have been shown to be site‐specific, reasonably efficient (40–49% in optimal constructs) [91] and with low‐level off‐target mtDNA mutagenesis. There were concerns over potential nuclear off‐target mutagenesis with the early editors [94]; however, additional engineering of the constructs has been able to alleviate this. At this time, there are no reports of the use of these very new base editors in cancer research; however, we predict that they will be an important tool for future studies. One thing of note regarding the use of cell models in cancer research is that the interaction of malignant cells with the tumour microenvironment (TME) is critical to tumour survival; therefore, this should be taken into consideration when interpreting the results of such studies.

### 3.3 Elucidating the functional effects of mtDNA mutations in cancer

#### 3.3.1 Complex I mtDNA alterations

Since over 50% of the mitochondrial genome encodes proteins of complex I, simple probability dictates that random mutational events affecting mtDNA are most likely to occur within complex I genes. The main function of complex I is to catalyse the transfer of electrons from nicotinamide adenine dinucleotide (NADH) (generated through the TCA cycle and fatty acid oxidation) to ubiquinone while simultaneously translocating four protons from the mitochondrial matrix into the intermembrane space. This stoichiometry has been widely accepted as a ratio of 4H^+^/2e^−^ [95]; however, a more recent re‐evaluation of the literature suggests a lower ratio of 3H^+^/2e^−^ is more likely [96]. In addition to the proton translocation by complexes III and IV, this creates the electrochemical gradient necessary for ATP synthesis [97]. Additionally complex I is the primary source of mitochondrial ROS [98] and is thus involved in maintaining cellular redox balance which has implications for cell survival and proliferation.

Tumours derived from cybrids harbouring a heteroplasmic m.12417insA frameshift mutation in *MT‐ND5* showed increased levels of mitochondrial ROS coupled with enhanced apoptotic resistance and increased tumour growth in comparison to their homoplasmic counterparts [99]. Further studies using the same heteroplasmic cybrids revealed a significantly increased level of Akt protein phosphorylation, a component of the potent PI3K‐Akt pro‐survival pathway which is frequently hyperactivated in human cancer (reviewed in ref. [100]). Importantly, the introduction of the yeast NADH quinone oxidoreductase (*NDI1*) gene into those cells restored complex I function and reversed the enhanced tumourigenicity. Similarly, *MT‐ND6* nonsense and missense mutations in lung adenocarcinoma cybrids also activate the Akt pathway in a ROS‐dependent manner [101]. A significant correlation between missense and nonsense *MT‐ND6* mutations and pathological grade, tumour stage and lymph node metastasis were reported in human lung adenocarcinoma samples. Furthermore, the migratory distance of the lung adenocarcinoma cybrids was significantly greater in those with *MT‐ND6* nonsense and missense mutations, suggesting the promotion of migration and invasion by these mutations [101]. The induction of epithelial‐to‐mesenchymal‐transition (EMT) is an important step in the progression of epithelial‐derived carcinomas to metastasis (reviewed in ref. [102]). Correlated with a poor prognosis, the upregulation of EMT is also associated with downregulation of OXPHOS in multiple cancer types [103].

In osteosarcoma cybrid cell lines in which a homoplasmic m.3571insC mutation in *MT‐ND1* completely disabled complex I activity, NADH accumulation was detected, inhibiting the activity of α‐KG dehydrogenase of the TCA cycle [104, 105]. This perturbed the α‐KG to succinate (SA) ratio in favour of α‐KG causing HIF‐1α (hypoxia inducible factor) destabilization. The stabilization of HIF‐1α is an important event in tumour progression, allowing tumour cells to adapt to the reduced availability of oxygen by upregulating glycolytic genes [106] and downregulating mitochondrial oxygen consumption [107]. Thus, m.3571insC reduced the tumourigenic potential of the osteosarcoma cells, while allotopic expression of wild‐type *ND1* rebalanced the α‐KG/SA ratio, restored HIF‐1α stability and sustained tumour growth. In contrast, homoplasmic *MT‐ND1* mutations conferring a milder effect on complex I activity (m.3460G>A/*MT‐ND1*) did not induce HIF1α destabilization or inhibit tumour growth [85]. Accordingly, these observations have led to the understanding that while defects that exert mild effects on complex I may have little or no impact on tumourigenicity, those that are severe may in fact be detrimental to tumour growth. Further studies have suggested that activation of adaptive mechanisms is also at play [108] and that nuclear encoded complex I deficiency may inadvertently promote tumour cell fitness by virtue of its ‘buffering’ by hypoxia [109], further stressing the complex web of molecular interactions in tumourigenesis.

In our own work, we recently demonstrated that preexisting age‐related mtDNA mutations resulting in complex I deficiency can accelerate intestinal tumour growth in *Apc* knock‐out mice [110]. *Lgr5‐creER;Apc* ^*fl/fl*^ [111] mice were crossed with the *PolγA* ^*mut/mut*^ [34, 35] mice (described in Section 2). Mice with age‐related mitochondrial dysfunction amassed a significantly greater intestinal adenoma burden resulting in a shortened lifespan compared with *PolγA* wild‐type mice. The predominant OXPHOS defect in the intestinal adenomas of *PolγA* ^*mut/mut*^ mice was complex I deficiency. Multi‐omics analysis revealed upregulation of the *de novo* serine synthesis pathway and mitochondrial one‐carbon metabolism in both the nontransformed epithelial crypts prior to induction and in the adenomas of 6‐month‐old *PolγA* ^*mut/mut*^ mice, implicating these pathways in the promotion of tumour growth. The importance of serine and one carbon metabolism in cancer cells is well established; these metabolic pathways are vital to the provision of nucleotides, antioxidants and anabolic precursors essential for maximal tumour growth (reviewed in ref. [112]).

There is currently only one tumour type in which mtDNA mutations have been shown to be oncogenic in the absence of nuclear drivers. Combined mtDNA and nuclear genetic analysis of renal oncocytomas has shown that the most frequent genetic events are loss of function mutations in complex I, with only a subset of those tumours exhibiting chromosome 1 loss and/or cyclin D expression [113]. These data are consistent with complex I mutations being tumourigenic driver events followed by nuclear mutations in this disease setting.

#### 3.3.2 Complex III mtDNA alterations

Complex III, also known as ubiquinol: cytochrome *c* oxidoreductase, is composed of 11 subunits, one of which, *MT‐CYB*, is encoded by the mitochondrial genome. Complex III catalyses electron transfer from ubiquinol to cytochrome *c*. This is coupled with the transfer of two protons across the inner mitochondrial membrane (IMM) [114]. In both xenograft and human models of bladder cancer, *MT‐CYTB* mutations have been shown to increase the generation of ROS and induce NF‐kB2‐signalling‐mediated tumour growth [115]. Similarly, the forced overexpression of a 7‐amino acid deletion mutation of *MT‐CYTB* increased ROS production and resistance to apoptosis in a transformed human uroepithelial cell line [116]. Complex III mutations have also been implicated in the promotion of an alternative metabolic pathway, namely, reductive carboxylation, within the context of OXPHOS‐deficient tumours. In contrast to canonical TCA, which metabolizes glucose and glutamine‐derived carbon in an oxidative manner, within reductive carboxylation, key reactions are reversed, and glutamine is used to generate TCA intermediates and acetyl‐coA, thus supporting lipid [117], protein and nucleotide [118, 119] synthesis reductively. This ensures provision of biosynthetic precursors essential to fuel the rapid growth of tumours and may be of particular importance in those tumours where oxidative capacity may be compromised by defects in TCA enzymes or components of the mitochondrial electron transport chain [120]. Using metabolomics and stable isotope tracing in a human osteosarcoma cell line containing a mutation in the complex III *MT‐CYB* gene, it was demonstrated that reductive carboxylation first requires the oxidation of αKG by αKG dehydrogenase generating NADH. The formation of NADPH from NADH by nicotinamide nucleotide transhydrogenase (NNT) reduces the NADP+/NADPH ratio driving the reverse reductive reaction of NADPH‐dependent IDH1 and IDH2 in the cytosol and mitochondria, respectively [120, 121].

#### 3.3.3 Complex IV mtDNA alterations

Complex IV is composed of 14 subunits (3 mtDNA encoded) and represents the final enzyme of the mitochondrial respiratory chain (reviewed in ref. [122]). An increased risk of ovarian cancer has been associated with mutations in the *MT‐CO1* gene [123], while the recent Pan‐Cancer Analysis of Whole Genomes (PCAWG) Consortium report *MT‐CO1* as the most frequently mutated mtDNA protein‐encoding gene in breast, cervical and bladder cancers [80]. Mutations within *MT‐CO1* have also been described as important contributors in the aetiology of prostate cancer. Within 260 prostate cancer samples, 12% of patients were found to harbour *MT‐CO1* missense mutations, while only 1.9% of 54 prostate cancer‐negative controls demonstrated mutations of this type. Sequencing of *MT‐CO1* identified three novel somatic amino acid changing mutations: m.5949G>A, m.6124T>C and m.6924C>T [124]. Generation of m.6124T>C mutant osteosarcoma 143B cybrids yielded tumour cells with a partial reduction in complex IV activity and increased generation of mitochondrial ROS and nitric oxide. Increased cellular proliferation and decreased apoptosis were observed *in vitro*, with an increase in tumour growth in immunodeficient mice also noted [125].

Mitochondrial OXPHOS subunit protein levels and enzyme activities were investigated in nine adenomatous polyps, 26 adenocarcinomas and their patient‐matched normal mucosa. The authors showed that 44% of the adenomas and 46% of the adenocarcinomas had decreased levels, or absence, of the MT‐CO1 subunit of complex IV compared with an average of 10% of normal crypts. These observations suggest that preexisting COX defects in normal colonic crypts may provide a selective metabolic advantage as they are enriched during colorectal carcinogenesis [110].

#### 3.3.4 Complex V mtDNA alterations

Complex V, also known as F_O_F_1_ ATP synthase, is comprised of 16 subunits, two of which (*MT‐ATP6* and *MT‐ATP8*) are encoded by the mitochondrial genome. Nonsynonymous mutations in these genes have been identified in several cancer types including thyroid [126], breast [127] and pancreatic [128] cancer and in osteosarcomas [129]. However, there is evidence of selection against truncating complex V mtDNA mutations, suggesting that some level of complex V function is required for cell survival [21]. The introduction of pathogenic m.8993T>G and m.9176T>C point mutations in the *MT‐ATP6* gene in to EB8 HeLa‐derived cell lines increased cell proliferation in culture and led to a greater expansion of tumour cells in xenografts, which was suppressed by the restoration of wild‐type *MT‐ATP6*. A reduced apoptotic index was demonstrated in the *MT*‐*ATP6* mutant cybrids both *in vitro* and *in vivo* in comparison to those bearing wild‐type mtDNA [130]. A similar study was carried out with the cytoplasmic transfer of the m.8993T>G mutation into the PC3 prostate cancer cell line. When transplanted into nude mice, tumours that developed from the mutant cybrids were seven times larger and generated significantly greater quantities of ROS than those that originated from wild‐type cybrids [124].

A more recent study carried out in yeast also investigated the effect of tumour‐specific mitochondrially encoded complex V mutations. The introduction of two mutations, Atp6‐P163S and Atp6‐K90E, found in prostate and thyroid tumour samples, respectively, was shown to increase the sensitivity of yeast cells to compounds inducing oxidative stress and also delayed the activation of the yeast permeability transition pore (yPTP) upon calcium induction, which may be of importance in resistance to apoptosis [131]. Interestingly, an earlier study carried out by the same group investigated the functional consequences of four cancer‐associated *mATP6* missense mutations (m.8914C>A, m.8932C>T, m.8953A>G and m.9131T>C) with only one (m.8932C>T) having a significant impact on mitochondrial function [132]. Taken together, the results of these studies suggest that while some complex V mutations may aid tumour cell resistance to apoptosis, mutations that significantly compromise mitochondrial ATP synthesis would have severe metabolic consequences for the tumour cell and therefore may be presumed intolerable.

#### 3.3.5 mtDNA copy number alterations

While specific mtDNA mutations and their relative proportions have been described extensively in relation to mitochondrial disease, ageing and cancer, it is becoming apparent that absolute mtDNA copy number is also an important factor in cancer pathology. Just as the occurrence and consequences of mtDNA mutations differ between different tumour types, changes in mtDNA copy number also vary and reports detailing their significance are also heterogeneous. For example, in comparison to adjacent normal tissue, breast, kidney clear cell carcinoma, hepatocellular carcinoma and myeloproliferative tumours demonstrate a reduced tumour mtDNA content, while lung, pancreatic and lymphocytic leukaemias display an increased mtDNA copy number [80]. However, when the presence and type of mtDNA mutations are considered, kidney chromophobe and thyroid tumours with mtDNA null allele fixations display much higher mtDNA copy numbers than those tumours without null allele fixations [81]. This suggests positive selection of an advantageous mutation within these tumours or a compensatory upregulation of mitochondrial mass in an attempt to counteract the effects of truncating mutations [80].

In the context of nuclear driver mutations, an upregulation of tumour mtDNA copy number has been significantly associated with *IDH1* mutation in low‐grade gliomas and *TP53* mutations in ovarian carcinoma. Conversely, the mutation of phosphatase and tensin homolog (*PTEN*) within low‐grade glioma exhibits the opposite pattern, driving a reduction in mtDNA abundance [133]. Mitochondrial metabolism and ROS generation are described as essential for *KRAS*‐induced cell proliferation and tumourigenesis with loss of *TFAM*, required for mtDNA replication and maintenance, disrupting mitochondrial function and consequently reducing tumourigenesis in a *Kras* driven mouse model of lung adenocarcinoma [134]. Conversely, the activation of *K‐ras* ^*G12V*^ in a HEK293 cell line led to a striking reduction in mitochondrial respiratory chain activity via the disruption of complex I. A subsequent elevation in glycolytic activity and ROS generation led to the increased tumourigenicity of transformed cells [135]. Although mitochondrial dysfunction in terms of defective OXPHOS has been associated with increased levels of apoptosis in ageing tissues [35, 136], diminished OXPHOS may have differing consequences following oncogene activation. Indeed, in mtDNA‐deficient *p* ^*0*^ 143B osteosarcoma cell lines in which *MYC* expression is reportedly high [137], the absence of functional OXPHOS and ensuing reduction in IMM potential (Δψm) were associated with the reduced activation of caspase 3 and an enhanced resistance to staurosporine‐induced apoptosis [138]. The evident interaction of mitochondrial function and mtDNA copy number with classical driver mutation‐mediated tumourigenicity suggests an intricate network of signalling pathways and dependences are at play and thus requires further investigation.

### 3.4 mtDNA alterations and clinical phenotype

Despite a significant body of literature investigating the effect of mtDNA mutations on tumourigenesis at the cellular level, the consequences of mitochondrial dysfunction at various stages of tumour progression and metastasis and how these align with the clinical phenotype are largely unknown. A recent study utilizing existing exome sequencing data reported a significantly greater survival in 344 patients with stage 1–3 colorectal cancer harbouring truncating mtDNA mutations or nontruncating, nonsynonymous mutations in comparison to wild‐type samples [21]. Truncating mutations affecting complex I were reported in approximately 20% of cases and those affecting the remaining respiratory chain complexes accounted for roughly 5% of cases. Additionally, a weak association between mitochondrial genotype and colorectal cancer consensus molecular subtype (CMS) [139] was observed with the canonical subtype CMS2 demonstrating some enrichment of mtDNA mutations [21].

The clonal and tumour‐specific nature of the expansion of mtDNA mutations has been proposed as a tool to assist in the differential diagnosis of gynaecological tumours. The correct diagnosis of the synchronous or metastatic nature of endometrial and ovarian cancers is of paramount importance as patient management and therapeutic regimes differ when tumours arise independently or via metastatic dissemination. As histological assessment can often yield inconclusive diagnoses and molecular screening can be laborious and expensive, mtDNA genotyping has been shown to provide a cost‐effective, easily implemented aid in the identification of synchronous versus metastatic endometrial and ovarian tumours [140, 141]. The same methodology has since been applied to breast and borderline ovarian tumours. In both cases, this has allowed the distinction between synchronous tumours and metastases, which have originated from the primary tumour, by virtue of whether or not they share a common mtDNA genotype [142, 143].

### 3.5 Can we exploit mtDNA alterations and OXPHOS defects to stratify patient treatment?

It is becoming apparent that a great metabolic heterogeneity exists among different tumour types and the contribution of OXPHOS function to tumourigenesis cannot be generalized. Depending upon tumour type and stage, mtDNA alterations may elicit tumour promoting or tumour inhibiting effects. As an acceptance is established that mtDNA alterations are a common feature of cancers, current focus is beginning to shift towards therapeutics and the implications that perturbations of mitochondrial function may have for treatment efficacy. Regarding the response to anticancer agents, mitochondrial OXPHOS defects, particularly those involving complex I, have been shown to modulate the apoptotic response to several chemotherapeutic agents. Following treatment with the DNA‐damaging agents etoposide and doxorubicin, caspase activation was significantly reduced in a complex I‐deficient cell line in comparison to wild‐type cells, thus reducing the chemotherapeutic response. In contrast, administration with the histone deacetylase inhibitor apicidin resulted in enhanced caspase activation in both complex I and grossly OXPHOS‐deficient cells in comparison to wild‐type cells, sensitizing them to treatment. [144].

When considering the therapeutic targeting of tumour metabolism, consideration of metabolic intratumour heterogeneity is crucial. A glycolytic tumour bulk phenotype with small populations of cancer stem cells reliant on mitochondrial OXPHOS has been described within several tumour types including pancreatic, colonic and ovarian cancers [145, 146, 147, 148]. Importantly, such OXPHOS‐dependent cancer stem cells are often resistant to standard therapies. Accordingly, a highly potent and specific small‐molecule inhibitor of mitochondrial complex I (IACS‐010759) has been shown to reduce tumourigenicity in models of acute myeloid leukaemia (AML) [149], in which cells are particularly dependent on OXPHOS [150, 151]. As glycolytic utilization of glucose is a principal compensatory adaptation to the inhibition of OXPHOS, IACS‐010759 efficacy was augmented under conditions of glucose restriction *in vitro* and in glycolysis‐deficient tumours *in vivo* [149]. Similarly, the glucose dependency of *Pten* ^*−/−*^ *;Trp53* ^*−/−*^ prostate cancer cells has also been exploited in conjunction with mitochondrial complex I inhibition, as demonstrated by the loss of selective killing of *Pten* ^*−/−*^ *;Trp53* ^*−/−*^ cells by the complex I inhibitor deguelin [152]. The IACS‐010759 complex I inhibitor has further been shown to reduce cell viability in adult T‐cell acute lymphoblastic leukaemia (T‐ALL) cell lines and a patient‐derived xenograft model, specifically in the context of oncogenic NOTCH driven OXPHOS reliance. The addition of IACS‐010759 to standard T‐ALL chemotherapies further compounded this effect, and although a compensatory utilization of glutamine in TCA cycle fuelling was observed, the use of the glutaminase inhibitor CB‐839 synergistically reduced T‐ALL cell viability in all models [153]

The anticancer effects of small molecule inhibitors of mitochondrial transcription (IMTs) targeting human mitochondrial RNA polymerase (POLRMT) have more recently been investigated as alternatives to the direct inhibition of OXPHOS [154]. Again, exploiting the OXPHOS dependence of cancer cell populations, the inhibition of cell growth was demonstrated in numerous cancer cell lines. Importantly, not all cell lines were responsive to IMTs, and resistance was shown to develop via a loss of genes intrinsic to mammalian target of rapamycin complex 1 (mTORC1) and von Hippel–Lindau (VHL) pathways in addition to an increased expression of mtDNA [155].

## 4 Concluding remarks

**Fig. 3:**
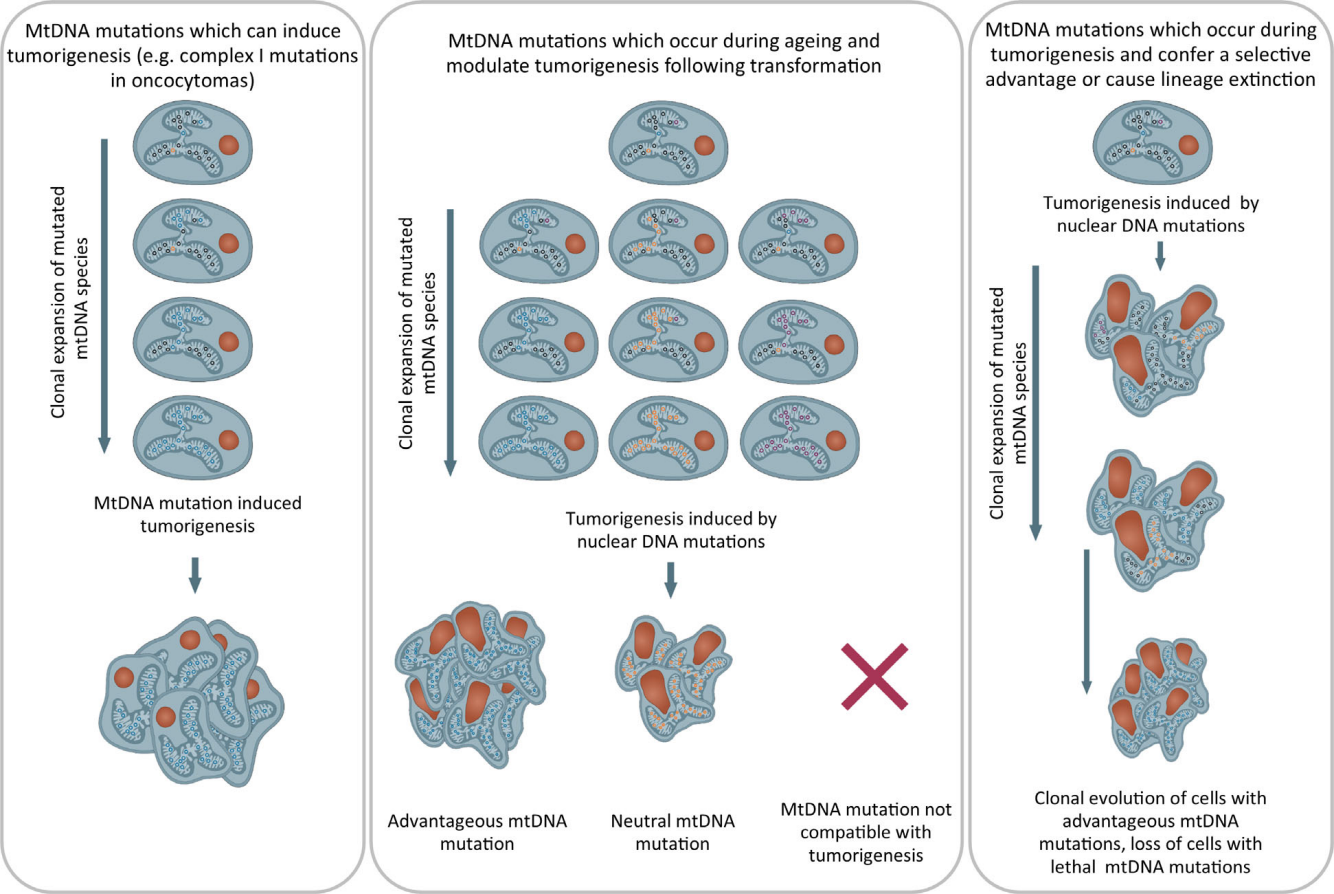
Hypothesized mechanisms by which mtDNA mutations may contribute to tumorigenesis. Three proposed mechanisms by which mitochondrial DNA (mtDNA) mutations may contribute to tumourigenesis. (1) mtDNA mutations can induce tumourigenesis directly followed by mutations to the nuclear genome as has been shown for renal oncocytomas. (2) mtDNA mutations occur and clonally expand over time until a biochemical oxidative phosphorylation (OXPHOS) defect and metabolic rewiring occur. Following oncogenic transformation through nuclear DNA mutations, mtDNA mutations may (i) provide a favourable metabolic environment for growth, provide resistance to therapy or enhance metastatic potential, (ii) be neutral and have no effect on tumourigenesis and (iii) not be compatible with tumourigenesis and cause cell death. (3) Low‐level mtDNA mutations which occur before or after transformation can clonally expand very rapidly due to the high rates of cell division within the tumour. mtDNA mutations with favourable effects on tumourigenesis could cause clonal evolution of that lineage, whereas lineages containing mtDNA mutations that do not confer a favourable phenotype could become extinct. [Colour figure can be viewed at wileyonlinelibrary.com]

The emerging picture highlights the wide variability in metabolic demand in different tissues and different cancer types, and therefore, the impact of mtDNA mutations on cancer development is likely to be equally dependent on the metabolic demands of the particular cell type. For example, in renal oncocytomas, complex I mutations can be drivers of tumourigenesis [113], whereas in other tissues such as the colon, they are unlikely to be drivers but may modulate tumourigenesis [110], and depending on the effect of the mutation on OXPHOS, this could be growth stimulatory or inhibitory [21]. In addition, the point of mtDNA mutation origin is unknown, and in many age‐related cancers, high‐level heteroplasmic or homoplasmic mtDNA mutations may be present long before the cell is transformed. Alternatively, due to accelerated clonal expansion afforded by rapid mitotic segregation, it is equally possible for mtDNA mutations to clonally expand during tumourigenesis and cell lineages in which they confer a favourable metabolic phenotype to outcompete neighbouring clones to become the dominate lineage within the tumour (Fig. 3).

Targeting of OXPHOS is becoming an attractive chemotherapeutic option in addition to standard treatment regimens. However, the heterogeneous nature of cancers must be taken into consideration as not all tumours, nor cancer stem cells, will necessarily respond in the same way to OXPHOS targeting, particularly if they have different metabolic capacities due to different mtDNA mutations. When considering targeting OXPHOS in cancers, a one size fits all approach should not be taken. Rather a prior determination of tumour OXPHOS status may assist in predicting patient response to a particular treatment regimen, enhancing patient stratification and personalized medicine, thus improving patient prognosis.

## Conflict of interest

The authors declare no conflict of interest.

## Author contributions

ALMS, JCW and LCG contributed to drafting the manuscript. LCG drafted the figures. All authors critically revised the manuscript and approved the final version.

## Data Availability

There is no data in this paper, it is a literature review.
